# A first principles study of convection cells to shear flow instability in 2D Yukawa liquids driven by Reynolds stress

**DOI:** 10.1038/s41598-025-87528-0

**Published:** 2025-01-27

**Authors:** Pawandeep Kaur, Rajaraman Ganesh

**Affiliations:** 1 Institute for Plasma Research, HBNI, Bhat, Gandhinagar, 382428 India; 2 Department of Chemical Physics, Weizmann Institute of Science, 7610001 Rehovot, Israel

**Keywords:** Fluid dynamics, Plasma physics, Physics, Nonlinear phenomena

## Abstract

The stability of kinetic-level convection cells (wherein the magnitude of macroscopic and microscopic velocities are of same order) is studied in a two-dimensional Yukawa liquid under the effect of microscopic velocity perturbations. Our numerical experiments demonstrate that for a given system aspect ratio $\beta$ viz., the ratio of system length $L_x$ to its height $L_y$ and number of convective rolls initiated $N_c$, the fate of the convective cells is decided by $\beta _c = \beta /N_c$. For $\beta _c < 1$, Reynolds stress is found to be self-consistently generated and sustained, which results in tilting of convection cells, eventually leading to shear flow generation, whereas for $\beta _c \ge 1$, parallel shear flow is found to be untenable. An unambiguous quantitative connection between Reynolds stress and the onset of shear flow using particle-level data is established without free parameters. The growth rate of the instability, the role of frictional forces, generalization of our findings and the possibility of realizing the same in experiments are also discussed.

## Introduction

Convection cell, an example of pattern formation in far-from-equilibrium systems^1^, is ubiquitous in nature and can be found in a wide variety of systems ranging from small scale systems such as tea kettel to large scale systems such as Sun’s surface^2^, oceans^3^ etc. One can also find its relevance in climate studies and in several technological applications such as solar devices, fusion reactors^4^ etc. Due to their ubiquity, in the past, extensive studies on instability of convective cells to shear flows have been conducted from hydrodynamic convective cells^5–8^ to Rayleigh-Bénard systems in fluids^9–12^ to Rayleigh-Bénard cells in Complex plasmas^13^ to Tokamaks^14^. Examples of several hydrodynamic studies in this direction are: generation of shear flows from convective cells by Finn et al.^5,10^ and Drake et al.^6^, mean or zonal flows in Tokamak plasmas during L to H mode transition^14^, experimental study of Rayleigh-Bénard convection cells by Krishnamurti and Howard^11^.

In spite of such extensive studies, an unambigous, first principles-based, quantitative proof on the mechanism of generation of shear flows has been lacking. In this work, we solve this outstanding problem without resorting to any free parameters. For this purpose, we choose strongly correlated Yukawa liquids (made up of charged grains immersed in a background plasma medium), which are often used as test beds to study fluid flow phenomena at microscopic or kinetic level^15,16^. A 2D convective flow is considered in Yukawa liquid confined in a semi-periodic domain as our starting point and its stability is investigated under microscale velocity perturbations using “first principles”classical molecular dynamics simulations. Our results demonstrate that for certain cell aspect ratio, convection cells become unstable and give rise to a vertically sheared mean flow (called bi-directional shear flow hereafter). The mechanism of generation of such flows is explained with the help of Reynold stress, constructed using particle data obtained from our simulations. The present work is first ever demonstration, wherein the microscopic details of the shear flow generation from kinetic-level convective cells is reported using molecular dynamics simulations.

## Simulation details

**Fig. 1:**
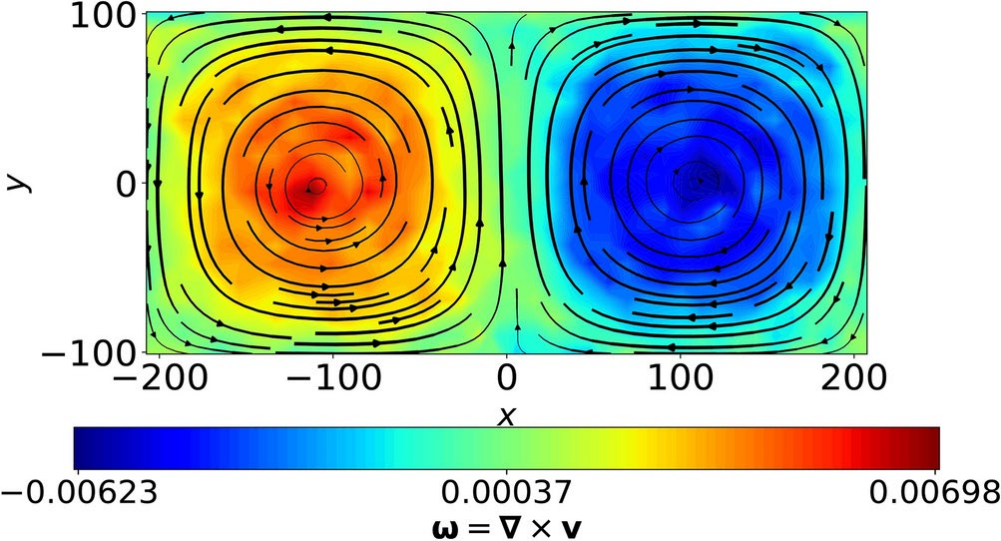
Convection cells obtained at $t = 1000\omega _{pd}^{-1}$ in a $\beta =2$ ($L_x = 2L_y \simeq 400a_0$) system of Yukawa liquid made up of $N =$ 28, 000 particles, where $\Gamma = 50$ and $\kappa =4$. The aspect ratio of each convective cell, $\beta _{c} = \beta / N_{c}$, where $N_{c}$ is the number of cells in a given geometry (Here, $N_c= 2$, hence $\beta _c=1$). The streamlines are obtained using time-averaged (averaged over $1000 \omega _{pd}^{-1}$) local fluid velocities, $\varvec{V}$ and the colorbar indicates the local magnitude of fluid vorticity, $\varvec{\omega } = \varvec{\nabla } \times \varvec{V}$ obtained by fluidization of grains over $(40 \times 20)$ grid.

Using multi-potential molecular dynamics code (MPMD-v2.0)^17^, we simulate *N* charged grains in 2D $x-y$ plane, wherein the *x* boundaries are periodic and *y* boundaries are specularly reflecting and it is assumed that $\partial /\partial z \equiv 0$. The intergrain interactions are modelled using screened Coulomb or Yukawa potential: $U = Q_d \exp (-r/\lambda _D)/(4\pi \epsilon _0 r)$, where $Q_d$ is the average charge of a grain, *r* is the intergrain distance and $\lambda _D$ is the effective Debye screening due to background electrons and ions. For the present study, the Complex plasma parameters such as coupling parameter, $\Gamma = Q_d^2/4\pi \epsilon _0 a_0 k_B T_d$ ($T_d$ is dust temperature and $a_0$ is Wigner-Seitz radius) and screening parameter, $\kappa =a_0/\lambda _D$ are tuned to lie in liquid regime on the phase diagram of Complex plasmas^18^, for example, we use $\Gamma = 5, 10, 20, 50, 75, 100, 500, 750, 1000, 1200, 1500$ for a given $\kappa =4$. The equations of motion are defined in non-dimensional form, where the length and time scales are normalized with respect to $a_0$ and inverse Complex plasma frequency, $\omega _{pd}^{-1}$ respectively. As our system size is large, we neglect the Ewald sums and truncate the pair-interactions for $r > 10a_0$^19^. Using Gaussian thermostat, the system is first thermalized at a desired temperature *T*, where $T=1/\Gamma$. To obtain the convection cells in an equilibrated 2D bed of grains, a flow is superimposed on the individual grain velocities in the following way:

1 $$\begin{aligned} v_{x}^{\prime } = v_{x} + V_o \sin (\pi y/L_y) \sin (N_c \pi x/L_x) \end{aligned}$$

2 $$\begin{aligned} v_{y}^{\prime } = v_{y} + V_o \cos (\pi y/L_y) \cos (N_c \pi x/L_x) \end{aligned}$$

where $(v_x, v_y)$ and $(v_x^{\prime }, v_y^{\prime })$ respectively represent the components of the instantaneous grain velocities before and after imposing the convective flow, *x*, *y* are the corresponding components of the instantaneous position vector of grains and $L_x(L_y)$ represents system size along $\hat{x}(\hat{y})$ such that the system aspect ratio, $\beta = L_x/L_y$. The aspect ratio of each cell can be defined as $\beta _{c} = \beta /N_{c}$, $N_c$ is the number of cells in a given geometry, $\beta$. The amplitude of base flow is equal to the average thermal velocity of grains, i.e., $V_o \simeq v_{th} = \sqrt{2/\Gamma }$. Hence we dub this structure as “kinetic-level convection cell”. The average kinetic and potential energies per particle dynamically relax in response to the imposed flow, however, the total energy per particle remains conserved (upto order $10^{-6}$) as the system is free from external drives. The imposed convective flow with $N_c=2$ thermalises into a pair of convective cells, such that $\beta _c = 1$, as shown in Fig. 1, where the colorbar indicates the magnitude of local fluid vorticity, $\varvec{\omega } = \varvec{\nabla } \times \varvec{V}$.

For better visualization, streamlines are plotted over time-averaged local vorticity, $\varvec{\omega }$ in Fig. 1, which are obtained using interpolation of local fluid velocities, $\varvec{V}$. The fluid quantities are obtained using fluidization technique, which distributes the grain information over a $(m \times n)$ grid, where *m* and *n* respectively represent the number of cells along $\hat{x}$ and $\hat{y}$. One should note that though the fluid quantities shown in Fig. 1 are obtained over $(40 \times 20)$ grid, the results are found to be insensitive to different grid sizes, provided the number of particles in each cell is sufficiently large.

Since the amplitude of the imposed convective flow is equal to the thermal velocity of the grains, the maximum convective velocity observed in our system is of the order of thermal velocity of the grains. This is in contrast to the convection cells observed in conventional hydrodynamic flows such as in oceans, tropical cyclones or in atmosphere, where the maximum convective velocity can be orders of magnitude larger than the thermal velocity of the constituent particles^20^. As pointed out earlier, convection cells obtained in our system can also be regarded as “kinetic-level convection cells”. In the absence of any other perturbation, such kinetic-level convection cells in 2D Yukawa liquids are found to sustain for long periods of time.

To test the stability of the kinetic-level convection cells, we apply a shear-flow perturbation (see Appendix) on the grain velocities along with the convective flow at $t=0$, such that the net momentum in the system along the periodic direction remains negligibly small, i.e.,

3 $$\begin{aligned} v_{x,i}^{\prime } = v_{x, i} + A \sin (\pi y_i/ L_y) \end{aligned}$$

where $v_x$, $v_x^{\prime }$ respectively represent the particle velocities before and after imposing the perturbation, *A* is the amplitude of the perturbation ($A = 0.25V_o$). The amplitude, *A* can be positive or negative, however, we consider positive value of *A* for the present study.

## Results

**Fig. 2:**
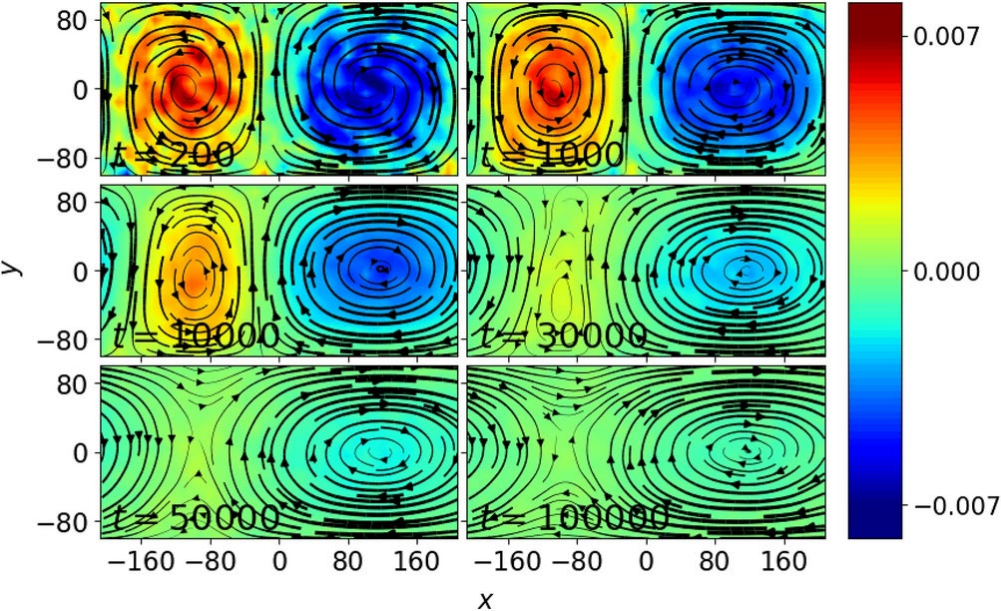
Snapshots of time-averaged local fluid velocity (streamlines), $\varvec{V}$ and vorticity (colorbar), $\varvec{\omega } = \varvec{\nabla } \times \varvec{V}$ for $\beta =2$ ($N_c = 2$, hence $\beta _{c}=1.0$) system of Yukawa liquid with $\Gamma = 50$ and $\kappa =4$ when subjected to particle-level velocity perturbation in addition to the convective flow. The fluid quantities are obtained using fluidization technique in a $(40 \times 20)$ grid and the time-average in each snapshot is performed over an indicated time period.

When perturbed (see Eq. 3), the resultant fluid flow undergoes a transition as illustrated in Fig. 2, where the streamlines and colobar represent the same quantities as in Fig. 1.

The fluid variables shown in Fig. 2 are time-averaged quantities obtained from particle-level data distributed over a $(40 \times 20)$ grid, where the average in each snapshot is performed over an indicated time-period. As the convective flow evolves, one of vortex starts growing in size at the expense of another vortex, thereby results in the emergence of a giant vortex with a separatix towards the end of $t = 50, 000\omega _{pd}^{-1}$ (It should be noted that the sign of *A* decides which vortex will shrink and eventually disappear in macroscale flow, for example, positive *A* results in disappearance of vortex with positive vorticity and vice-versa). The macroscale fluid flow does not evolve much beyond this stage, which is evident from the macroscale fluid flow profile at $t = 100,000\omega _{pd}^{-1}$.

**Fig. 3:**
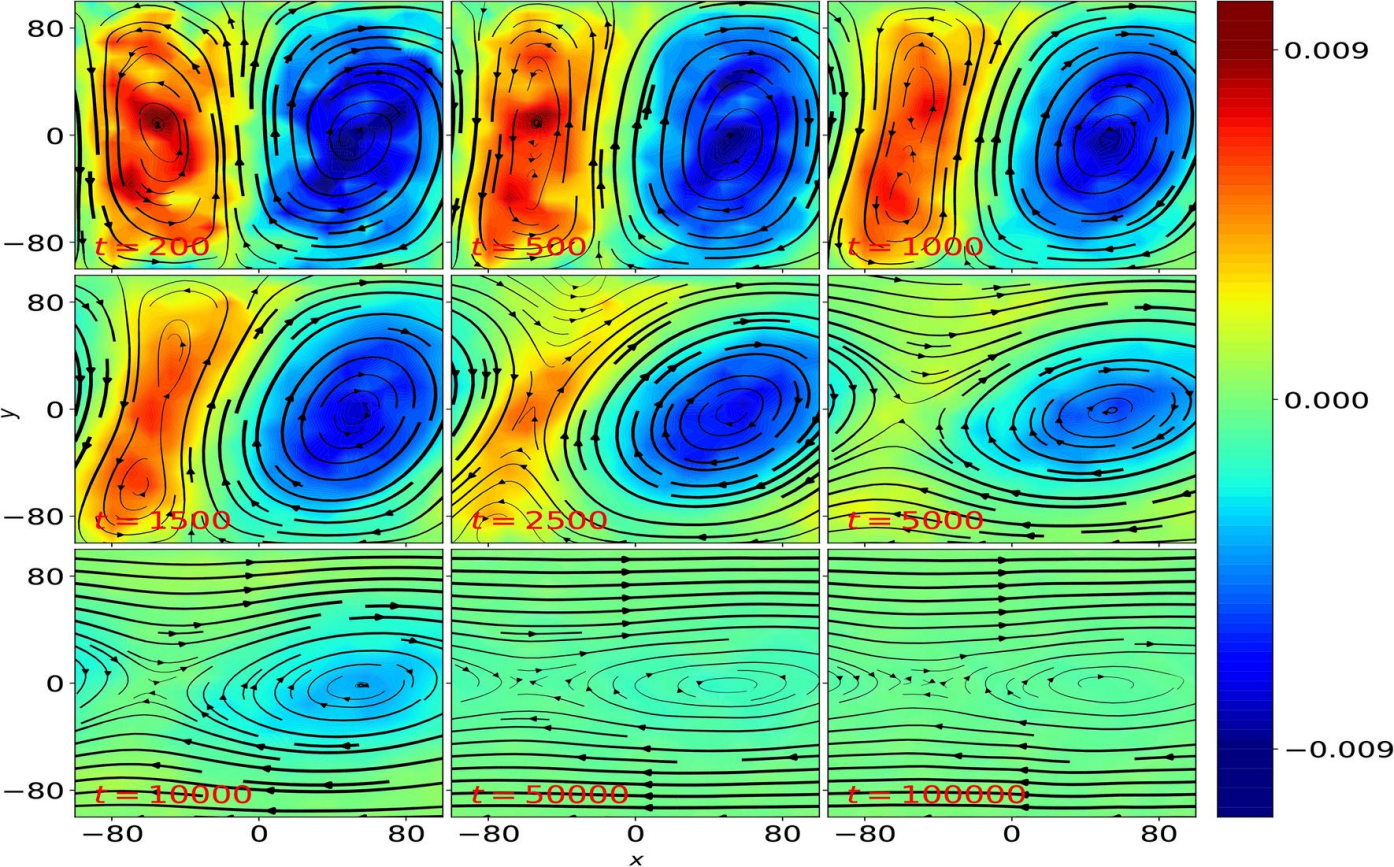
Time-averaged local fluid velocity (represented by streamlines) and vorticity (colorbar) profiles at various *t* values in a $\beta =1$ ($N_c=2$, hence $\beta _c=0.5$) system of Yukawa liquid with convective flow, when placed under the effect of velocity perturbation, where $\Gamma =50, \kappa =4$. In each snapshot, the local fluid quantities are defined over a $(20\times 20)$ grid and the time-average is performed over an indicated time period.

In order to test the role of cell aspect ratio on the stability of convective flow, we repeat the experiment for other $\beta _c$ values. For higher $\beta _c$ (i.e. $\beta _c \ge 1$), the fluid flow undergoes transitions similar to the case shown in Fig. 2, but does not result in a shear flow. However, this is not the case for systems with $\beta _c < 1$. For example, when an initial convective flow with $\beta _c=0.5$ ($N_c = 2$) is subjected to a velocity perturbation (as given in Eq. 3), it undergoes a series of transitions different from $\beta _c \ge 1$ case (see Fig. 3). A pair of convection cells is observed initially upto $t = 200\omega _{pd}^{-1}$, which then undergoes a tilting phase from $t = 500$ to $t = 10, 000\omega _{pd}^{-1}$, wherein convection cells are seen to disappear and a horizontal shear flow emerges in the system. After passing through several mixed states , where convective flow co-exist with shear flow (see $t = 2500, 5000, 10, 000\omega _{pd}^{-1}$), a bi-directional shear flow is seen to emerge towards the end of $t = 50, 000\omega _{pd}^{-1}$, where the fluid velocities are directed along opposite directions near the top and bottom walls. The direction of fluid flow near the top and bottom walls will be reversed for negative value of perturbation amplitude, *A*. Once the macroscale vortices completely disappear and a bi-directional flow is emerged in the system, the fluid flow does not evolve much, as can be seen at $t = 50, 000$ and $100, 000\omega _{pd}^{-1}$. As described earlier, the local fluid quantities are obtained using fluidization technique over $(20 \times 20)$ grid.

**Fig. 4:**
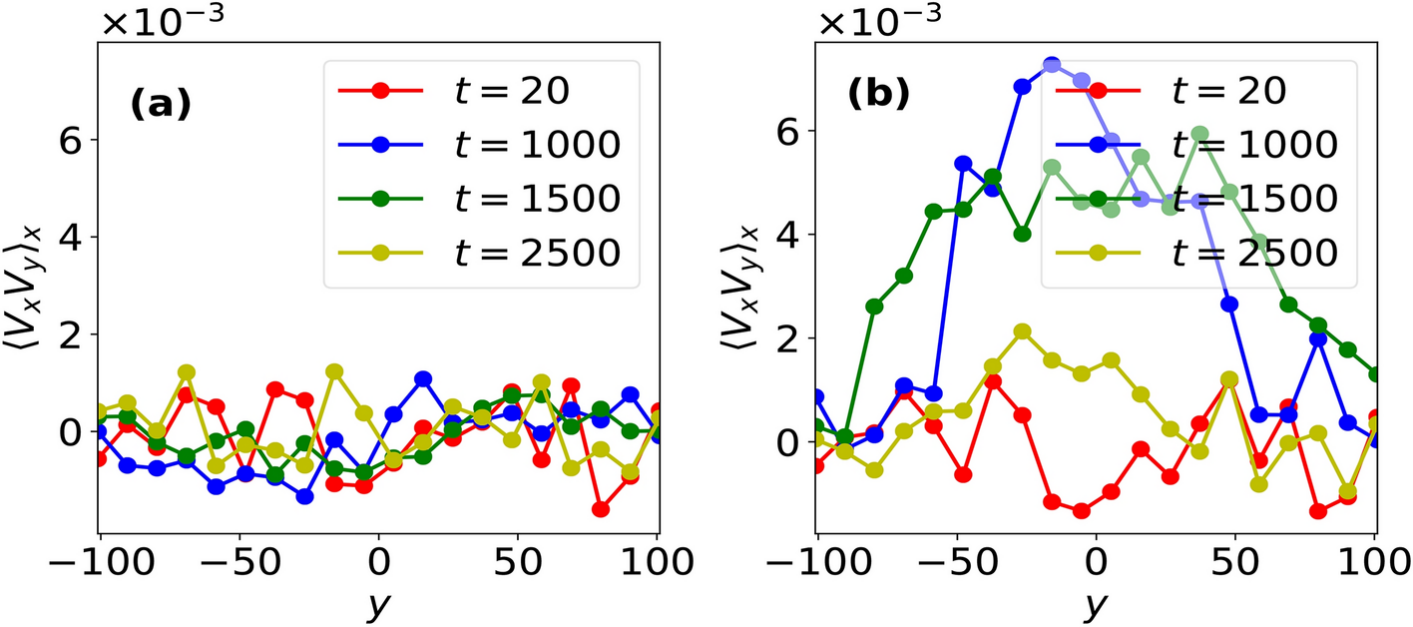
Averaged (*x*-averaged) correlation between horizontal and vertical components of the fluid velocities, $\langle V_x V_y \rangle _x$ as a function of vertical height *y* at various *t* values obtained in a convective flow placed under velocity perturbation of amplitude *A* given in Eq. (3) for $\beta =1$ system using (**a**) $A = 0.0$ and (**b**) $A = 0.05$.

In order to explain the mechanism which brings fluid flow transition from convective flow to bi-directional shear flows for $\beta _c < 1$, we calculate Reynolds stress^14,21^ using particle data obtained in our MD simulatons. The generation of shear flow via Reynolds stress is governed by the following^5,14,22^:

4 $$\begin{aligned} \frac{\partial }{\partial t}\langle V_x \rangle _x = -\frac{\partial }{\partial y} \langle \tilde{V}_x \tilde{V}_y \rangle _x \end{aligned}$$

where $\tilde{V}_x, \tilde{V}_y$ respectively represent fluctuation in fluid velocities along $\hat{x}$ and $\hat{y}$. For our system of Yukawa liquid composed of small number of grains, Reynolds stress is estimated through the variation of *x*-averaged correlation of horizontal and vertical components of fluid velocities, $\langle V_x V_y \rangle _x$ with vertical height, *y* as shown in Fig. 4. It can be seen from Fig. 4a that the velocities $V_x$ and $V_y$ are uncorrelated at all times in the absence of any velocity perturbations, thereby, Reynolds stress or the quantity $-\partial \langle V_x V_y \rangle _x /\partial y \sim 0$ and hence, the stability of convective cells is not affected in this case. On the other hand, a non-zero velocity correlation (and hence, Reynolds stress) is seen to emerge under the effect of velocity perturbation, which eventually generates a bi-directional shear flow in the system. This can be explained as follows: the velocity perturbation introduces an initial tilt in the convection cells as shown at $t = 500\omega _{pd}^{-1}$ in Fig. 3, which correlates the horizontal and vertical fluid velocities and hence, generate Reynolds stress in the system. The Reynolds stress thus generated results in titling of the cells, which further increases the magnitude of Reynolds stress. This feedback mechanism, i.e., Reynolds stress generation by tilting of cells, which in turn increases the tilt, leads to a positive reinforcement between the two quantities which continues to operate till a bi-directional shear flow unfolds in the system with the near disappearnce of convection cells. As a result, the fluid velocities $V_x$ and $V_y$ decorrelate and hence, Reynolds stress eventually becomes zero in the system (see $t = 2500\omega _{pd}^{-1}$ in Fig. 3), thereby changing the macrostate of the system irreversibly. To the best of our knowledge, this work is the first ever demonstration of kinetic-level convective cells undergoing a transition to shear flow via Reynolds stress dynamics obtained using particle-level Molecular Dynamics data.

**Fig. 5:**
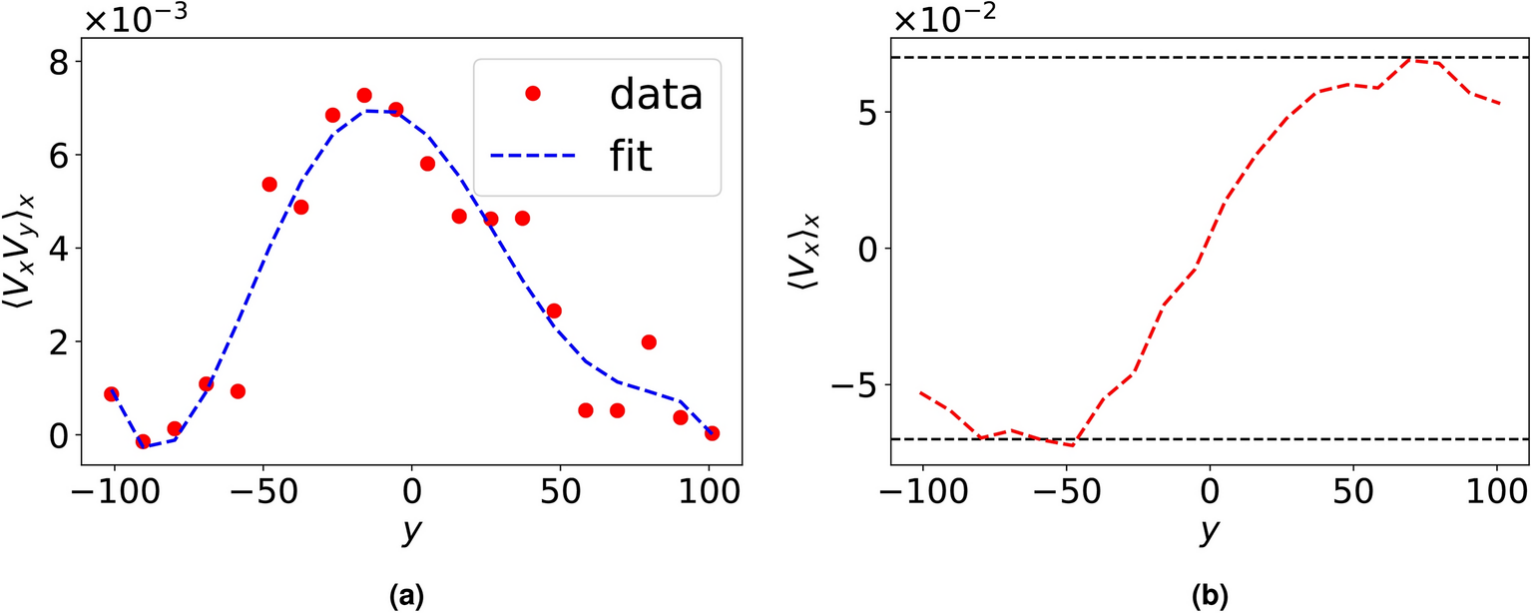
(**a**) Variation of *x*-averaged correlation of horizontal and vertical fluid velocities, $\langle V_x V_y \rangle _x$ and (**b**) *x*-averaged horizontal fluid velocity, $\langle V_x\rangle _x$ as a function of *y* at $t = 1000 \omega _{pd}^{-1}$. The dashed horizontal lines in (**b**) indicate the expected amplitude of horizontal velocity generation from Reynolds stress. The dashed line in (**a**) shows a polynomial fit over the data points.

We find that the Reynolds stress calculations performed using particle data correctly predicts both the magnitude and direction of bi-directional flow generated in our system. To demonstrate this, let us consider the velocity correlation profile at $t = 1000\omega _{pd}^{-1}$ (see Fig. 5a, wherein a polynomial fit is shown for visualization purpose. One can see that Reynolds stress, $-\partial \langle V_x V_y \rangle _x / \partial y$ is negative for $y < 0$, which using Eq. (4) gives $\partial \langle V_x \rangle _x /\partial t < 0$. Similarly, the direction of flow can be predicted for $y > 0$, which is along $+\hat{x}$. These directions of shear flow predicted using the slope of velocity correlation (or Reynolds stress) shown in Fig. 5a agrees well with the shear flow observed in the system (see Fig. 5b). Interestingly, the magnitude of the shear flow can also be predicted with the help of Eq. (4) as:

5 $$\begin{aligned} \langle V_x \rangle _x \simeq \langle V_x V_y \rangle _x\frac{\tau }{L} \end{aligned}$$

where *L* is the scale length of the velocity correlations, $\langle V_x V_y \rangle _x$ and $\tau \sim t$ is the time at which the magnitude of the flow $\langle V_x \rangle _x$ is to be predicted. From Fig. 5a $(\langle V_x V_y \rangle _x)_{max} \simeq 0.007$, $L \simeq 100a_0$ and $\tau \simeq 1000 \omega _{pd}^{-1}$, therefore the amplitude of the shear flow, $\langle V_x \rangle _x \simeq 0.07$, which comes out to be nearly same as observed in shear flow profile shown in Fig. 5b.

**Fig. 6:**
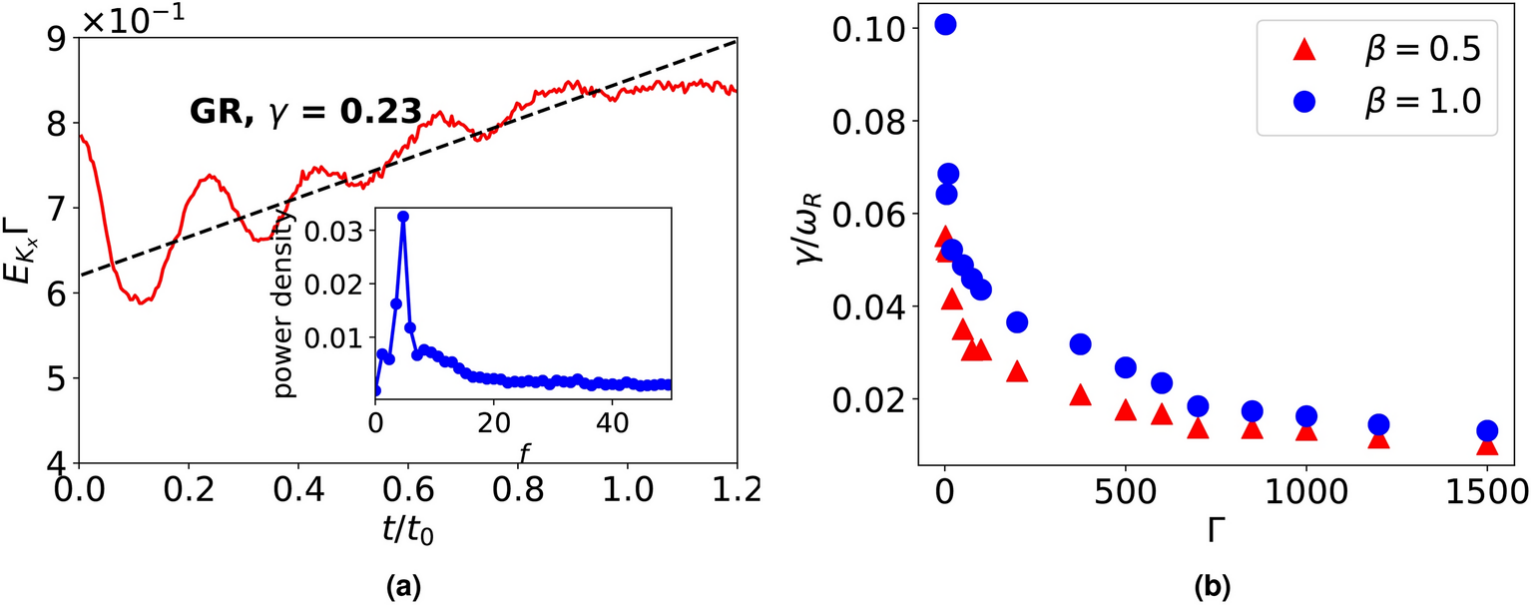
(**a**) Growth rate, $\gamma$ of normalized *x*-kinetic energy, $E_{K_x}\Gamma$ obtained in an initialized convective flow of Yukawa liquid ($\Gamma = 50, \kappa =4$) under velocity perturbation of amplitude, $A = 0.05$ in a $\beta =1$ system ($N_c=2$, $\beta _c=0.5$). The growth rate exhibits a real frequency as illustrated using Fourier transform of the growth rate (see inset), (**b**) shows the variation of normalized growth rate, $\gamma /\omega _R$ as a function of coupling strength, $\Gamma$ among the grains.

We calculate the growth rate of this kinetic-level shear flow instability for a range of coupling strengths, $\Gamma$ between the grains, since the viscosity of correlated liquids is controlled using $\Gamma$ values^23,24^. To find the growth rate for a given $(\Gamma , \kappa )$ pair, we plot horizontal kinetic energy, $E_{K_x}\Gamma$ (i.e., normalized with respect to thermal energy, $E_K =1/\Gamma$) as a function of time, $t/t_0$, where $t_0$ represents eddy turnover time defined as $t_0 = 2\pi R_{eddy}/v_{th}$ ($R_{eddy}$ is average radius of an eddy for a given $\beta$). The growth rate, $\gamma$ is obtained by fitting a straight line to the normalized *x*-kinetic energy as shown in Fig. 6a for $\beta =1$ system with $\Gamma =50$. The existence of periodic oscillations in normalized *x*-kinetic energy curve suggests the presence of real frequency, $\omega _R$, which can be found using Fourier transform of the normalised *x*-kinetic energy (see inset of Fig. 6a). Following the same procedure, growth rate and frequency can be found for other $\Gamma$ values in $\beta = 0.5$ and $\beta =1$ systems. We find that while the growth rate, $\gamma$ decreases with an increase in coupling strength $\Gamma$, between the grains, the real frequency, $\omega _R$ shows the opposite trend (Fig. 6b shows normalized growth rate $\gamma /\omega _R$ for a range of $\Gamma$ values). This reduction in growth rate, $\gamma$ may be attributed to higher spatial correlations for increasing $\Gamma$ values.

**Fig. 7:**
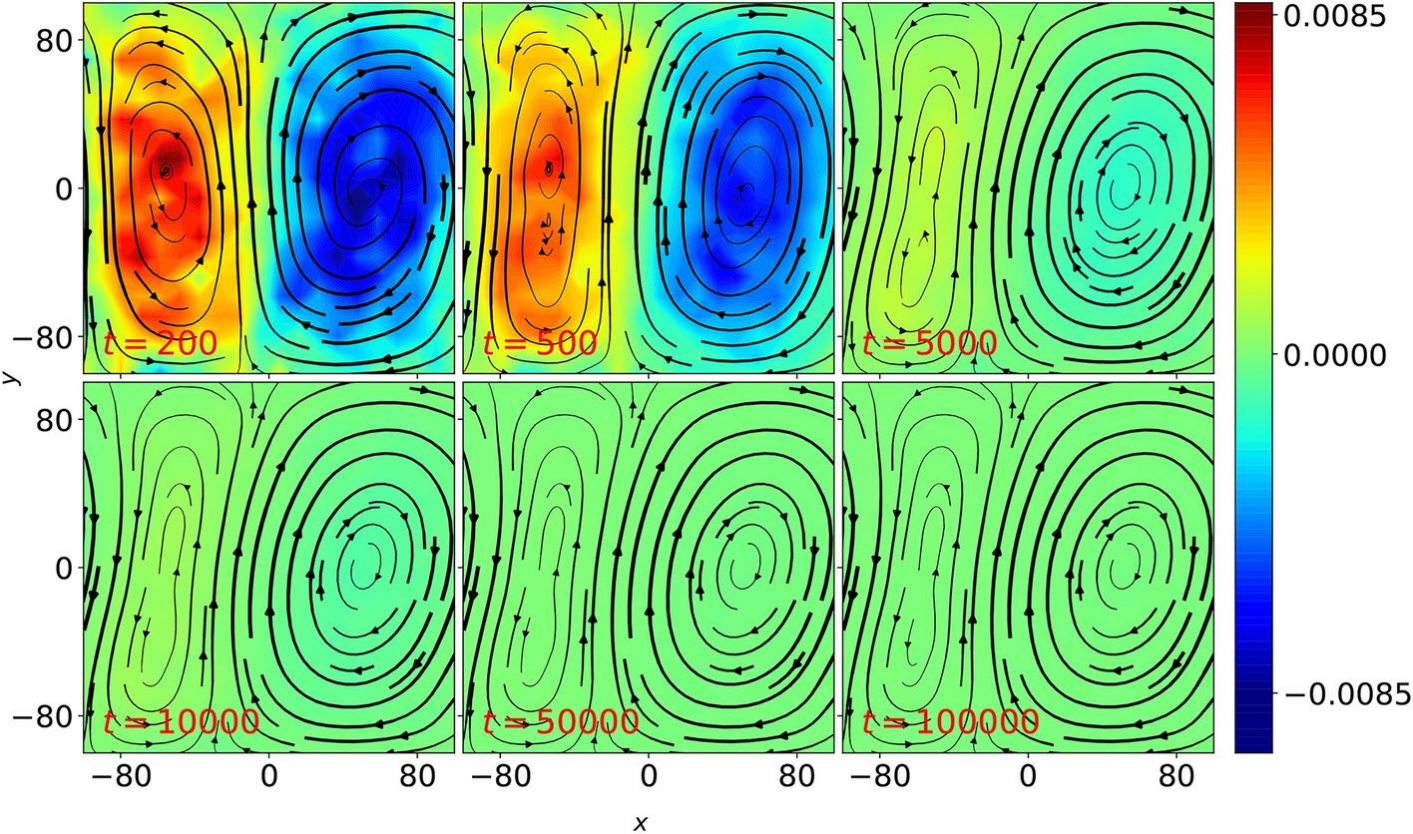
Time-evolution of fluid flow profiles represented by streamlines and vorticity (colorbar), $\varvec{\omega }$ for Yukawa liquid under the velocity perturbation of amplitude, $A = 0.05$ imposed over convective flow with $\beta _c < 1$, where dust-neutral collisions with collision frequency, $\nu _d = 0.001$ are also considered.

**Fig. 8:**
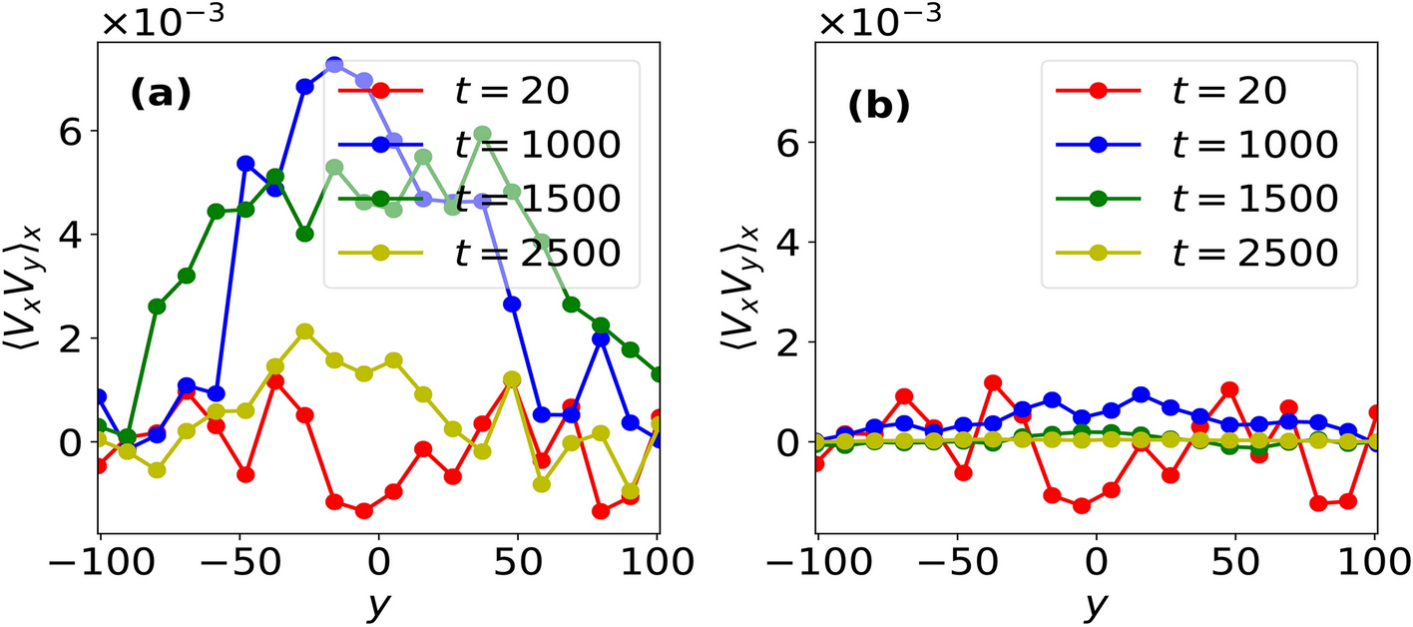
*x*-averaged correlation between horizontal and vertical components of fluid velocity, $\langle V_x V_y \rangle _x$ at various *t* values, when a velocity perturbation of amplitude $A = 0.05$ is imposed over convective flow in the (**a**) absence ($\nu _d = 0$) and (**b**) presence of dust-neutral collisions with frequency, $\nu _d = 0.001$.

Furthermore, we also investigate the stability of kinetic-level convection cells in the presence of frictional forces such as dust-neutral collisions, which are usually present in Complex plasma experiments. In the presence of such collisions, the equation of motion is modified as:

6 $$\begin{aligned} \ddot{\varvec{r}}_i = \varvec{f}_i - \nu _d \varvec{\dot{r}}_i \end{aligned}$$

where $\nu _d$ is the normalized collision frequency and $\varvec{f}_i$ is the force acting on *i*th grain due to all other grains. We find that while the velocity perturbation does tilt the macroscale convective cells, however, the presence of dust-neutral collisions suppress the generation of Reynolds stress. As a result, further tilting of convection cells and hence, the emergence of macroscale bi-directional shear flow is suppressed for $\beta _c < 1$ as shown in Fig. 7, where macroscale fluid flow does not evolve beyond $t = 5000\omega _{pd}^{-1}$ for $\nu _d = 0.001$, where the value of $\nu _d$ is obtained (in normalized units) using Complex plasma parameters using in laboratory experiments^25^. Figure 8 further illustrates the role of neutral collisions using Reynolds stress calculations, where unlike $\nu _d = 0$ case shown in Fig. 8a, velocity correlations, $\langle V_x V_y \rangle _x$ are suppressed for $\nu _d = 0.001$ (see Fig. 8b).

In the absence of collisions ($\nu _d=0$), the suppression of tilting instability and hence stability of convective cells for $\beta _c \ge 1$ can be understood via vertical advection of the mean flow by convective rolls^26^, which provides negative feedback to the bi-directional shear flow generation by Reynolds stress. For “flatter”cells i.e, $\beta _c \ge 1$ , the vertical advection effectively counters mean flow formation due to Reynolds stress, setting up a cutoff $\beta _c$ (here cutoff $\beta _c =1$).

## Discussion

We investigate the stability of an initialized pair of kinetic-level convection cells under microscale velocity perturbations in 2D Yukawa liquids using “first principles” classical molecular dynamics simulations. Our main findings are the following:

- We find that while a pair of kinetic-level convection cells merge to generate a giant vortex in large aspect ratio system ($\beta _c \ge 1$), whereas such convection cells in small aspect ratio systems ($\beta _c < 1$) show the emergence of bi-directional shear flow after passing through several intermediate states. This finding corroborates with the results obtained in previous 2D hydrodynamic studies performed in fluids obeying Navier-Stokes equations^5^.
- The mechanism of such shear flow generation starting from a pair of convection cells is analyzed via Reynolds stress calculations performed using particle data obtained from our simulations. We demonstrate that a finite Reynolds stress is generated in a system of convection cells under a class of microscale velocity perturbations, which tilts the convection cells and eventually leads to the generation of bi-directional shear flow for $\beta _c < 1$. There are no free parameters used.
- The growth rate, $\gamma$ of such instability is found to decrease with increasing $\Gamma$ values. A real frequency is observed, which increases with an increase in $\Gamma$ values.
- Furthermore, the stability of convective flow is also examined under the effect of frictional forces viz., dust-neutral collisions, where the collisions are found to suppress the macroscale shear flow generation in the system by inhibiting the Reynolds stress mechanism.

Finally, the use of Yukawa liquid presents us with a possibility of validating our findings in laboratory Complex plasma experiments, as one can track the positions of individual dust grains in such experiments with the help of relatively simple optical devices. For example, one can generate convection cells in Complex plasmas using an external drive^27^ and study their stability under microscale velocity perturbations. We believe that our findings would remain valid in a variety of systems where convection cells dynamically emerge under various external forces such as temperature or density gradient, gravity etc^28,29^.

## Figures and Tables

**Fig. 9:**
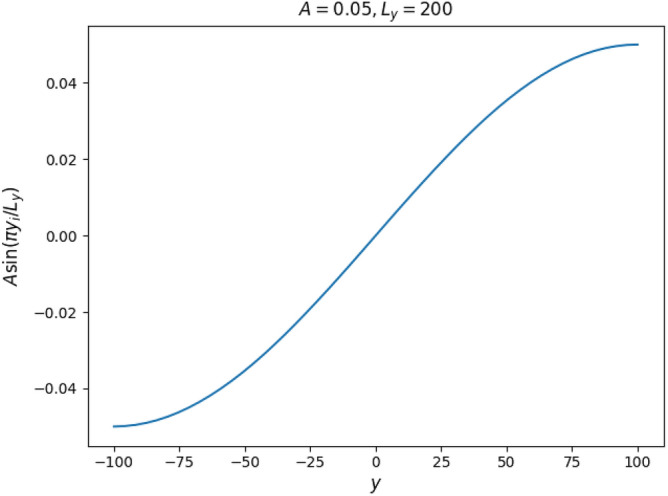
An example demonstrating shear flow velocity perturbation introduced in the system at a given *x* value for $A = 0.05, L_y=200$ ($-L_y/2<= y <=L_y/2$).

## Data Availability

Data is available upon request to the corresponding author.

## Appendix

Typical form of the velocity perturbation introduced in the system is shown in Fig. 9. Clearly, it is a shear flow perturbation as for a given point in *x*, the magnitude of added perturbation increases with vertical height *y*.
